# Daily‐Level Drinking Motives as Predictors of Transdermal Alcohol Concentration and Alcohol‐Induced Blackouts Among Young Adults

**DOI:** 10.1111/acer.70426

**Published:** 2026-10-06

**Authors:** Shannon D. Glenn, Michael A. Russell, Robert J. Turrisi, Veronica L. Richards

**Affiliations:** ^1^ Edna Bennett Pierce Prevention Research Center The Pennsylvania State University University Park Pennsylvania USA; ^2^ Department of Biobehavioral Health The Pennsylvania State University University Park Pennsylvania USA; ^3^ TSET Health Promotion Research Center, Stephenson Cancer Center University of Oklahoma Health Sciences Tulsa Oklahoma USA; ^4^ Department of Health Promotion Sciences University of Oklahoma Health Sciences Tulsa Oklahoma USA

**Keywords:** alcohol‐induced blackout, drinking motives, transdermal alcohol concentration; young adults

## Abstract

**Background:**

Alcohol use is necessary—but not sufficient—for understanding why individuals experience an alcohol‐induced blackout (AIB). Drinking motives are one of the most proximal risk factors for alcohol use. This study conducts a comprehensive within‐person examination of AIBs predicted by prospective daily reports of drinking motives and alcohol use measured using a transdermal alcohol concentration (TAC) biosensor.

**Methods:**

Participants were young adults who engaged in risky drinking and experienced at least 1 AIB in the past 3‐months (*N =* 169, *M*_*age*_ = 20.8, 53% female, 87% White, 10% Hispanic). They wore TAC biosensors and completed twice daily surveys about drinking motives and AIBs over six social weekends (i.e., Thursday–Sunday). TAC drinking features (peak, rise rate, and rise duration) were extracted from TAC data for each day. Two‐level multilevel structural equation models were conducted to examine the associations between drinking motives, latent intoxication estimated from TAC features, and AIBs. Aim 1 examined separate models for each motive (enhancement, social, coping, conformity). Aim 2 examined a single model with each motive adjusting for the others.

**Results:**

In Aim 1, on days when participants reported higher enhancement, social, coping, and conformity motives, the associated increase in TAC intoxication was associated with increased odds of experiencing an AIB. In Aim 2, when all motives were entered into a single model, only social motives resulted in increased TAC, which subsequently increased the odds of an AIB occurring.

**Conclusions:**

Findings demonstrate that days when an individual reports above average drinking motives relative to their own average, they are at increased risk of engaging in riskier drinking, which may result in subsequent AIBs. These findings suggest that daily changes in drinking motives may be an important intervention pathway for preventing risky drinking and subsequent AIBs.

## Graphical abstract

This study examined alcohol‐induced blackouts (AIBs) predicted by daily drinking motives (enhancement, social, coping, conformity) and transdermal alcohol concentration (TAC) intoxication. On days when individuals reported higher versus lower enhancement, social, coping, and conformity motives, they experienced riskier TAC intoxication, which subsequently increased the chances an AIB would occur (Aim 1). When all motives were entered as predictors in a single model, only social motives were associated with riskier TAC intoxication, which subsequently increased AIB odds (Aim 2).

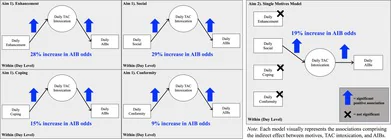

## 1 Introduction

It is estimated that one in four young adults ages 18–25 engaged in heavy‐episodic drinking in the past month (4+/5+ females/males; NSDUH 2025). This type of risky drinking increases individuals' immediate risk for experiencing an alcohol‐induced blackout (i.e., absence of memory for all or part of a drinking episode; Miller et al. 2018; Rose and Grant 2010). Approximately 4 in 5 young adults will experience an alcohol‐induced blackout (AIB) during college (Glenn et al. 2022) and many young adults experience recurrent AIBs (Barnett et al. 2014; Richards, Glenn, et al. 2023; Richards, Turrisi, et al. 2023; Richards et al. 2024). Studies examining college student and young adult samples with a history of AIBs found that 1 in 3 drinking episodes resulted in the occurrence of an AIB (Glenn et al. 2026; Richards et al. 2024). Experiencing an AIB exacerbates individuals' risk for experiencing other negative alcohol‐related consequences, includes more chronic or severe consequences like alcohol use disorder, sexual consequences, and driving under the influence (Glenn et al. 2023; Hingson et al. 2016; Linden‐Carmichael et al. 2023; Robertson et al. 2019; Studer et al. 2019; Valenstein‐Mah et al. 2015; Voloshyna et al. 2018; Wilhite et al. 2018). On days when an AIB occurs, individuals experience about 3.5 more negative alcohol‐related consequences relative to days when an AIB does not occur. Evidence from these studies suggest AIBs are a critical intermediary construct to reduce harm among young adults who engage in risky drinking.

Alcohol use is necessary but not sufficient for understanding why individuals experience an AIB (Glenn et al. 2023; Merrill et al. 2019). AIBs do not always happen after a certain number of drinks or at a specific intoxication level, even across two occasions within the same person. One of the most proximal risk factors of alcohol use is drinking motives (Cooper 1994). There is an extensive literature on the associations between drinking motives and alcohol use (Bresin and Mekawi 2021; Patrick and Terry‐McElrath 2021; Votaw and Witkiewitz 2021). There is also evidence suggesting that drinking motives are positively associated with experiencing AIBs (Merrill et al. 2019; Ward et al. 2023; Wetherill and Fromme 2016). The current study will expand on previous research by assessing day‐level prospective associations between drinking motives, alcohol use measured using a novel transdermal alcohol concentration (TAC) biosensor, and AIBs among young adults.

### 1.1 Incentive Motivation Model of Alcohol Use

The Incentive Motivation Model of Alcohol Use posits that an individual's motivational drive to drink alcohol is related to expected interoceptive changes produced by alcohol consumption (Cox and Klinger 1988, 2011). Drinking motives are categorized into four distinct subtypes, including: (1) enhancement (e.g., because drinking is exciting), (2) social (e.g., to celebrate a special occasion with friends), (3) coping (e.g., to forget about your worries), and (4) conformity (e.g., to help you fit in; Cooper 1994; Cooper et al. 1995; Cooper et al. 2016). The associations between drinking motives and alcohol use have been extensively studied at the person‐level using cross‐sectional and longitudinal methods (e.g., Boyle et al. 2022; White et al. 2016). Person‐level studies consistently provide evidence for a positive association between enhancement motives and alcohol use (e.g., Bresin and Mekawi 2021; Merrill and Read 2010; White et al. 2016). Some person‐level studies suggest social and coping motives are positively associated with alcohol use (e.g., Boyle et al. 2022; Cooper 1994; Schick et al. 2022; White et al. 2016) or that they may have no association with alcohol use (e.g., Merrill et al. 2014; Read et al. 2003). The majority of person‐level studies also provide support for a negative or null association between conformity motives and alcohol use (e.g., Bresin and Mekawi 2021; Cooper 1994; White et al. 2016).

Early work refining the Incentive Motivation Model suggested that drinking motives may not be stable and should be assessed repeatedly and close in time to a drinking episode (Cooper et al. 1995). Findings from studies at the day‐level (e.g., daily diary, ecological momentary assessment) have been both consistent and inconsistent with findings at the person‐level (Boyle et al. 2026; Dvorak et al. 2014; Hamilton et al. 2020; Patrick and Terry‐McElrath 2021; O'Donnell et al. 2019; Votaw and Witkiewitz 2021). For example, Dvorak et al. (2014) found that person‐level enhancement motives were associated with alcohol use, but day‐level enhancement motives were not. O'Donnell et al. (2019) found that day‐level enhancement motives predicted whether someone would initiate a drinking episode, but enhancement motives were not associated with continuing to drink. Similar to person‐level studies, day‐level findings also provide support for both positive (Hamilton et al. 2020; Howard et al. 2024; O'Hara et al. 2015) and null associations (O'Donnell et al. 2019; Patrick and Terry‐McElrath 2021) between social motives, coping motives, and alcohol use. Unlike findings at the person‐level, many day‐level findings provide evidence for a positive association between conformity motives and alcohol use (O'Donnell et al. 2019; O'Hara et al. 2015). These findings highlight differences in drinking outcomes based on whether motives are examined at the day‐level (i.e., how individuals change from day‐to‐day) or the person‐level (i.e., how individuals differ from each other on average).

### 1.2 Drinking Motives and AIBs

There is less known about the relationship between drinking motives and AIBs (Merrill and Read 2010; Merrill et al. 2014, 2016, 2019; Miller et al. 2020; Riordan et al. 2019; Shank et al. 2020; Ward et al. 2023). Merrill and Read (2010) conducted a cross‐sectional study among college students who drink alcohol and examined the associations between the 4 drinking motive subtypes, drinking, and 8 consequence domains (including AIBs). They observed a positive direct association between enhancement motives and AIBs, but no other significant direct or indirect associations were observed. In a follow‐up study, Merrill et al. (2014) examined how baseline drinking motives predicted drinking and AIBs at the 1‐year follow‐up. They found that baseline enhancement motives were positively associated with AIBs at follow‐up, through their indirect association with drinking. No other direct or indirect associations were observed between other motive subtypes, drinking, and AIBs. In a different longitudinal study, Merrill et al. (2016) examined latent growth classes of AIBs among college student drinkers with a pre‐college baseline survey and bi‐weekly surveys starting in the first week of college lasting throughout the first 2 years of college. They observed that students with higher enhancement motives upon entry to college were more likely to be in higher risk groups with consistent AIB experiences over time. They also observed that students with higher conformity motives upon entry to college were more likely to experience a decrease in the likelihood an AIB would be experienced over time. Shank et al. (2020) examined the 4 motive subtypes and AIBs once during the first or second year of college and again approximately five years post‐college. Enhancement and coping motives were positively associated with AIBs during college, controlling for drinking. At the 5‐year follow‐up, enhancement and conformity motives were positively associated with AIBs, controlling for drinking.

Few studies have assessed within‐person differences in drinking motives and AIBs. Ward et al. (2023) measured AIBs retrospectively once a week over 10 weeks to examine between‐ and within‐person differences between the 4 motives subtypes and AIBs among college women. They found that when students reported higher social, coping, enhancement, and conformity motives, they were more likely to experience an AIB than they were on days with lower levels of these motives, controlling for baseline drinking. When all motives were included in a single model, they found that only coping motives were associated with experiencing an AIB at the within‐person level, controlling for drinking. Merrill et al. (2019) conducted a 28‐day daily‐diary study examining retrospective reports of drinking, AIBs, and 5 drinking motive items among a sample of heavy drinking college students. Hierarchical linear modeling revealed no significant associations between the 5 drinking motive items and AIBs. These models controlled for daily drinking, however, which may have adjusted away a key mechanism leading from motives to AIBs. When each of the 5 motive items were tested in individual models with no covariates, only “to get high, buzzed, or drunk” (enhancement subtype item) significantly predicted the likelihood of an AIB. Findings from these studies suggest there is an association between drinking motives, drinking, and AIBs. However, study design (e.g., retrospective reporting of motives), samples with different risk profiles (e.g., female only, samples who engage in risky and non‐risky drinking), and measurement differences (e.g., not all studies examine all motives subtypes) make it difficult to draw definitive conclusions about these associations. Additional research assessing drinking motives at the daily‐level, prior to alcohol use occurring, is needed to assess the associations between the 4 motives subtypes, drinking, and AIBs among a sample of young adults who engage in risky drinking.

### 1.3 The Current Study

The current study will address gaps in the current literature by using intensive event‐level data to prospectively examine the four motives subtypes and their associations with subsequent drinking, and AIBs among a sample of young adults who engage in risky drinking. Drinking was measured with transdermal alcohol concentration (TAC) biosensors (the BACtrack Skyn). The use of TAC biosensors in the context of examining AIBs (i.e., partial or full memory loss) is advantageous for several reasons. Recall of self‐report drinking may be less accurate when risky drinking occurs (Northcote and Livingston 2011). It is therefore plausible that individuals may experience difficulties in recalling the number of drinks they consumed the day/night prior, especially if an AIB was experienced. TAC biosensors are not dependent on recall and thus provide a powerful alternative to drinking measurement in natural settings where AIBs occur. The biosensor measures TAC repeatedly to provide a curve of biological alcohol intoxication for each drinking day (Fridberg et al. 2022; Richards et al. 2024; Russell et al. 2022). In the current study, we focus on three TAC drinking features (referred to as TAC intoxication) derived from this curve that are indicative of risky drinking: (1) rise rate, the rate of TAC increase during a drinking event (analogous to the speed of alcohol consumption), (2) peak, the maximum level of TAC during a drinking event (analogous to the intensity/amount of alcohol consumption), and (3) rise duration, the number of hours spent under rising TAC during an event (analogous to the amount of time spent consuming alcohol; Russell et al. 2025). This will be the first study to examine AIBs, drinking measured using a TAC biosensor, and prospective reports of drinking motives among young adults who engage in risky drinking. The study hypotheses focus on examining associations at the daily‐level (within‐person) rather than average differences (between‐person) to answer why and when AIBs occur (which event‐days result in an AIB vs. not). Within‐person findings are further complemented by using a TAC biosensor to precisely assess drinking at the daily‐level. Key study aims and hypotheses are described below.

#### 1.3.1 Aim 1

The first aim of the study was to test the raw, unadjusted associations of drinking motives reported on drinking days with TAC intoxication and AIBs. Separate models were conducted for each drinking motive to test its raw association with TAC intoxication (i.e., latent intoxication factor estimated from three TAC drinking features) and AIB risk (see Figure 1). Consistent with previous literature (e.g., Hamilton et al. 2020; Howard et al. 2024; O'Donnell et al. 2019; O'Hara et al. 2015), it was hypothesized that motives would be positively associated with TAC intoxication (H1a, *path a2*). Based on previous work (e.g., Shank et al. 2020; Ward et al. 2023), it was also hypothesized (H1b) that motives would be positively associated with AIBs (*path c2*). In each model, indirect effects were tested to assess TAC intoxication as a key mechanism linking motives to AIBs (*paths a2 x b2*). It was hypothesized (H1c) that drinking motives would be indirectly positively associated with AIBs, through its association with TAC intoxication.

**FIGURE 1:**
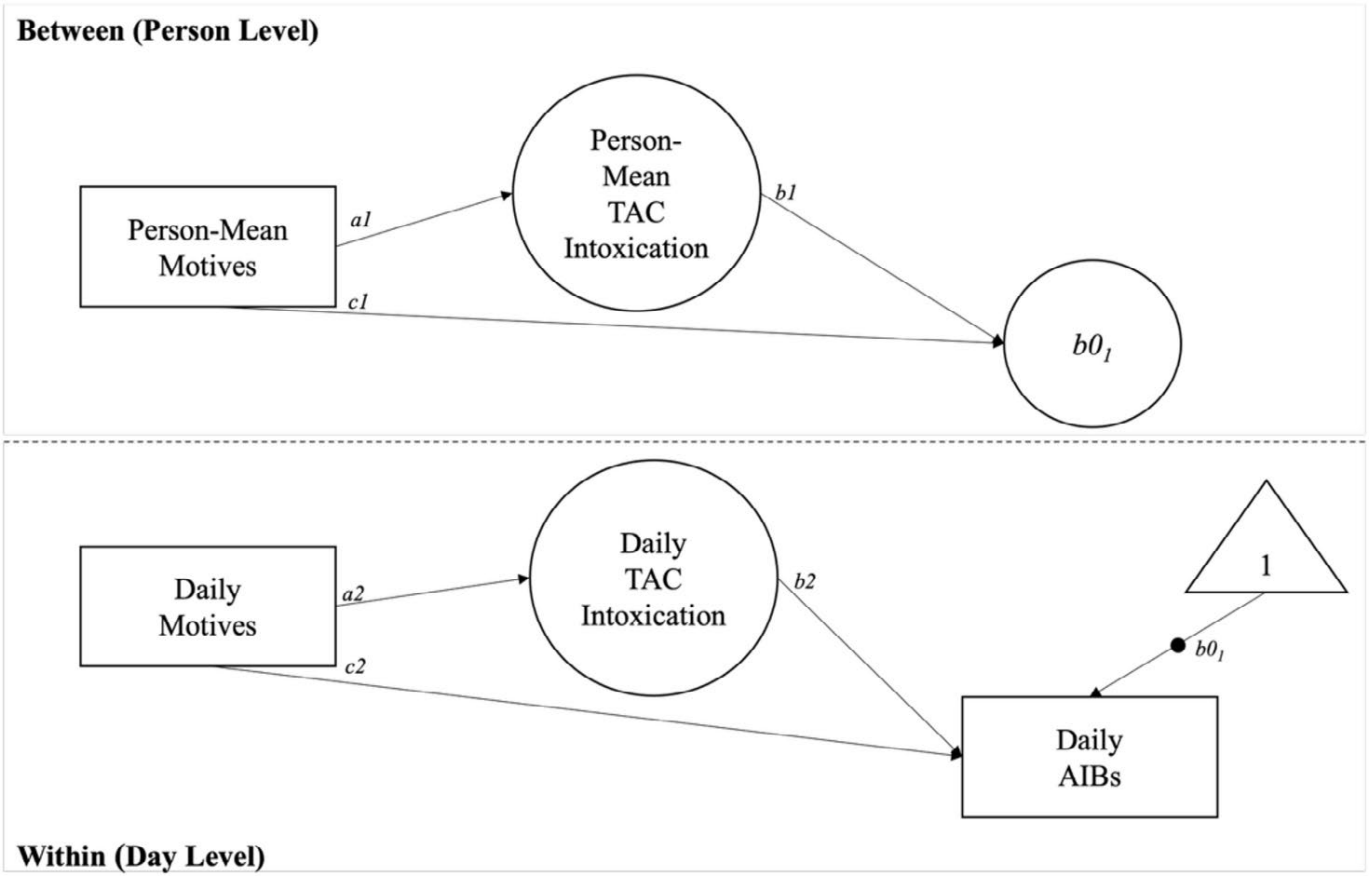
Theoretical multilevel structural equation model presenting hypothesized direct and indirect paths For Aim 1 linking drinking motives, TAC intoxication, and AIBs.

#### 1.3.2 Aim 2

The second aim of the study was to test the independent contributions of drinking motives reported on drinking days with TAC intoxication and AIBs by examining each motive subtype as a separate predictor in a single model (see Figure 2). This approach tests the contributions of each motive subtype, adjusting for the others. For example, this tests whether enhancement motives are associated with AIBs among young adults who report the same amount of social, coping, and conformity motives, and provides an estimate of how much information is offered by each motive subtype in the prediction of AIBs. Hypotheses are consistent with Aim 1. It was hypothesized that each of the drinking motives subtypes would be independently and positively associated with TAC intoxication (H2a, *path a2*) and AIBs (H2b, *path c2*). It was also hypothesized (H2c, *paths a2 x b2*) that drinking motives would be indirectly positively associated with AIBs, through its association with TAC intoxication.

**FIGURE 2:**
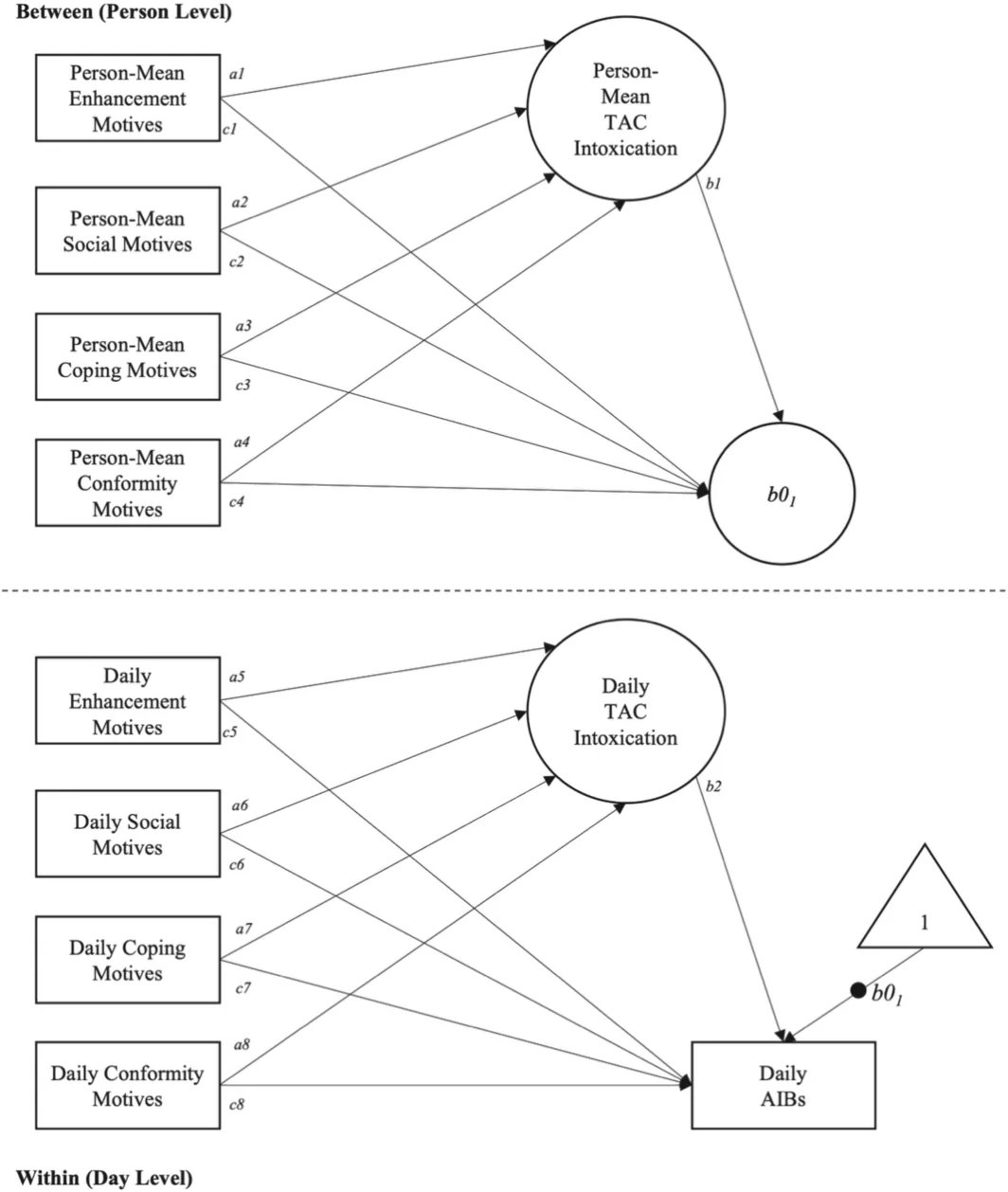
Theoretical multilevel structural equation model presenting hypothesized direct and indirect paths for Aim 2 linking drinking motives, TAC intoxication, and AIBs.

## 2 Materials and Methods

### 2.1 Recruitment Procedures

Detailed methods are published elsewhere (Glenn et al. 2026). Young adults from a large, public university and surrounding area in the northeastern United States were recruited. Recruitment methods included email invitations to randomly selected students from the registrar's database and flyers on and off campus. Participants were emailed an invitation describing the study which included a personalized URL to access a screening survey. Eligible participants were immediately routed to a 20‐min baseline survey. After completing the baseline survey, participants scheduled a 20‐min enrollment visit appointment to come into the laboratory to receive the alcohol biosensor and training on the study protocol. The final enrolled sample included 175 participants. All procedures were approved by the University's Institutional Review Board and all participants provided informed consent.

### 2.2 Data Collection Procedures

Data collection occurred in the Spring of 2025 and spanned 6 social weekends (i.e., Thursday to Sunday) across two bursts (3 social weekends per burst). There was a one‐month break between Burst 1 and Burst 2 (retention between burst 1 and 2: 90.9%). During each burst, participants were instructed to wear the BACtrack Skyn biosensor on their wrist continuously from Thursday evenings at 5:00 p.m. through Sunday morning at 9:00 a.m. They were instructed to remove the biosensor to shower or if they were engaging in other activities that would get the biosensor wet. At the end of each social weekend (i.e., Sunday morning at 9:00 am), participants were asked to sync and upload their TAC data and to power off and charge the device prior to the next social weekend. Participants also received a morning (open from 9:00 am to 4:00 pm) and evening (open from 4:00 pm to 7:00 pm) survey each Thursday, Friday, Saturday, and Sunday (48 daily surveys total; 83.9% completion rate). Average completion time for the morning surveys was 7.4 min (SD = 21.7 min) and 3.4 min (SD = 11.3) for evening surveys. The average time from survey open to submission for the morning survey was 10:53 a.m. (M = 113 min, SD = 99.59) and 4:45 p.m. for the evening survey (M = 45 min, SD = 46.28). They also initiated ecological momentary assessments (EMA) when starting their first alcoholic drink and continued to receive hourly follow‐ups until they were no longer drinking. Participants were compensated up to $225 via Amazon electronic gift cards or money deposited to the University payment system.

### 2.3 Measures

#### 2.3.1 Standard Drink Count

On each morning and evening survey participants were asked, “How many standard drinks of alcohol did you have yesterday? A standard drink is a 12‐ounce beer (a 24‐ounce beer = 2 standard drinks), a 5‐ounce glass of wine, or a shot of liquor.” Response options ranged from 0 (no drinks consumed) to 30 (30+ drinks consumed). This measure was used to prompt follow‐up survey questions (i.e., AIBs) and to identify the analytic sample (this is discussed more in the results section paper).

#### 2.3.2 Current Drinking

On each morning and evening survey, participants were asked, “Have you had any drinks of alcohol today?” Response options were dichotomous, with 0 (no) and 1 (yes). This measure was used to create the drinking motive composite variables and for sensitivity analyses on the AIB outcome (see analytic plan for more detail).

#### 2.3.3 Alcohol‐Induced Blackouts

AIBs were measured using 8 items from The Alcohol‐Induced Blackout Measure‐2 (ABOM‐2; Boness et al. 2022), adapted for daily use. These items were assessed in the morning and evening surveys after drinking was self‐reported on the Friday, Saturday, and Sunday morning or evening surveys (standard drink count > 0). The morning assessment was designed to measure AIB consequences that participants immediately remembered upon taking the survey. It is possible that a participant may not remember an AIB consequence until later in the (e.g., “able to remember a small part of the day after being reminded,” “reminded about things you had previously forgotten”). The evening assessment was designed to increase sensitivity of the AIB measure by asking participants if they recall any additional AIB consequences from the previous drinking episode. In the morning survey, participants were asked, “As a result of drinking yesterday, did you… (insert AIB item)?” In the evening survey, participants were asked, “Since your morning report, did you find out or remember that as a result of drinking yesterday you… (insert AIB item)?” Response options for each item were dichotomous with 0 (no) and 1 (yes).

Three composites were created from the eight items. First, if a participant endorsed any of the 8 items, they were considered to have experienced an AIB that day. The AIB outcome was used for the study's main hypotheses. Separate composites were then created to distinguish by type of AIB. Fragmentary AIBs included 4 items: “unable to remember a few minutes,” “reminded about things you had previously forgotten,” “have fuzzy memories of events,” “and able to remember a small part of the day after being reminded.” En bloc AIBs included 4 items: “wake up with no idea where you had been,” “unable to remember hours at a time,” “suddenly realize you have no memory,” and “unable to remember what happened the night before.” If a participant endorsed any of the 4 fragmentary AIB items, they were considered to have experienced a fragmentary AIB. If they endorsed any of the 4 en bloc AIB items, they were considered to have experienced an en bloc AIB. The fragmentary and en bloc AIB outcomes were used in supplementary analyses.

#### 2.3.4 Drinking Motives

Drinking motives were assessed on the Thursday, Friday, and Saturday morning and evening survey using 20 items from the Drinking Motives Questionnaire‐Revised (DMQ‐R; Cooper 1994). Participants were asked, “Right now how motivated are you to drink (insert motive item)?”. Response options were on a 3‐point scale ranging from 0 (no), 1 (somewhat), and 2 (definitely). Separate composites were created for morning and evening assessments for each motive subtype. Each composite was averaged separately for morning and evening assessments and included 5 items for enhancement, (e.g., “because you like the feeling”), 5 items for social (e.g., “to be sociable”), 5 items for coping (e.g., “to forget about your worries”), and 5 items conformity motives (e.g., “to be liked”). Omega values for each subscale were high (ω between‐person ranged from 0.97 to 0.99; ω within‐person ranged from 0.94 to 0.98).

The study goals were to assess changes in within‐person motives from day‐to‐day and not variations in within‐person motives throughout the day. Consistent with other EMA studies, it was decided to create a single composite item that was an average of morning and evening motives (e.g., Howard et al. 2024; O'Donnell et al. 2019). Prior to data analyses additional work was completed to achieve an analytic sample that consisted of individuals reporting motives prior to alcohol use occurring. Data cleaning focused on Thursday, Friday, and Saturday morning and evening reports (*n* = 3150 total days). First, on the morning and evening survey, participants were asked, “Have you had any drinks of alcohol today?”. Days when participants did not report drinking during the morning or evening survey did not need any additional cleaning (*n* = 2269, 72% of total days). These reports were averaged to create a single daily motives score. Days when individuals reported already initiating a drinking episode on the morning survey were coded as missing (*n* = 38, 1.3% of total days). Days when participants did not complete a morning survey but reported drinking on the evening survey were also coded as missing (*n* = 2, 0.1% of days). Individuals who did not report a drinking episode on the morning survey but did report drinking on the evening survey had their evening motives scores recoded to missing (*n* = 222, 7% of days). Rather than drop these days from the analytic sample it was decided to only use the morning motives scores for these individuals. Thus, no average score was computed. The same process was followed for those with missing data. For individuals who completed the morning survey but not evening (*n* = 233, 7.4% of days) or completed the evening and not the morning (*n* = 341, 10.8% of days), only 1 score was used to represent that day.

#### 2.3.5 Transdermal Alcohol Concentration Drinking Features

Procedures for processing TAC data have been published previously (Richards et al. 2024; Richards and Russell 2025; Russell et al. 2025). The current study focused on 3 TAC drinking features: rise rate (the rate of alcohol consumption), peak (the intensity or amount of alcohol consumption), and rise duration (the amount of time spent consuming alcohol) (Richards et al. 2024; Russell et al. 2022, 2025). TAC data was segmented into “social days” because drinking often extends past midnight and does not fit neatly into a midnight‐midnight calendar day. The boundary for the end of a social day aligned with the end of the timing of the morning survey at 9:00 a.m. TAC drinking features were extracted from each social day with TAC positive data. Episodes containing multiple TAC episodes were calculated using all data for the social day. Each individual feature was operationalized according to previously published guidelines (Russell et al. 2025): Rise rate was the average of increasing point‐to‐point TAC rates (+TAC/h) for each day. If multiple TAC episodes were present, rise rate was averaged using data from all episodes for the social day. Peak was the maximum TAC observed for the day's drinking event(s). Rise duration was the total number of hours in which the TAC curve was rising that day. If multiple TAC episodes were present, the total number of hours was calculated using data from all episodes for the social day. TAC drinking days were those with at least one drinking episode identified (i.e., positive TAC data). TAC non‐drinking days were those with no identified drinking episodes but with at least 80% of biosensor wear for the social day since this suggested no drinking occurred. TAC features for non‐drinking days were recoded to 0. Features were left missing if no episodes were present, but the biosensor was worn for less than 80% of hours of the social day (i.e., 9:00 a.m.–9:00 a.m.).

The hypotheses and subsequent analyses examine a latent factor for TAC intoxication which includes indicators for rise rate, peak, and rise duration. These three features represent the rising portions of TAC curves, which represent biological intoxication trajectories. The latent variable derived from these features can be thought of as a biological intoxication composite. This is supported by strong and positive standardized loadings for each feature onto the latent biological intoxication factor (see Table 2). A latent variable was also used to facilitate parsimony and reduce the number of models estimated.

#### 2.3.6 Control Variables

Sex at birth was coded as male (0) and female (1). Data collection burst was coded as burst 1 (0) and burst 2 (1). Participants were asked on the Friday, Saturday, and Sunday morning survey if they used cannabis the previous day. Cannabis use was coded as no previous day use (0) and previous day use (1).

### 2.4 Data Analytic Strategy

Multiple sets of analyses were conducted prior to testing the study hypotheses. First, sensitivity analyses were conducted in MPlus version 8 (Muthén and Muthén 2017). Three path models were examined to test whether current drinking was associated with experiencing any type of AIB the day prior, fragmentary AIBs, or en bloc AIBs. Second, unconditional “empty” multilevel models examining each drinking motive as an outcome were run in R 4.3.2 using the lmer function to generate means, variances, and intraclass correlations (ICCs) (R Core Team 2023). Third, a measurement model was conducted to examine the unconditional fit to the data of two‐level models representing the latent within‐ and between‐subject factor of TAC intoxication. Rise rate, peak TAC, and rise duration were partitioned into their between‐ and within‐person components prior to model estimation using R 4.3.2. These variables were then z‐scored to allow for comparison of effect sizes. The multilevel measurement model was then examined in MPlus version 8 using Bayesian estimation. A latent factor representing TAC intoxication was specified at the within‐ and between‐person levels with indicators for rise rate, peak TAC, and rise duration. Peak TAC was identified at both levels by fixing the loading to 1. Rise rate and rise duration were estimated freely at both levels. Traditional SEM fit statistics are not available when examining multilevel models with Bayesian estimation; we therefore rely on standardized factor loadings and the indicator of R^2^ to assess model fit.

Analyses to test the study hypotheses were conducted in MPlus version 8. Five multilevel structural equation models (ML‐SEM) were conducted to examine the primary outcome of whether any AIB occurred. Each ML‐SEM used Bayesian estimation with non‐informative priors and were specified according to guidelines for when mediating variables vary at both the between‐ and within‐person level (e.g., Fang et al. 2019). Each model was specified according to the following: Drinking motives and TAC drinking features were daily‐level predictors that varied both within‐ (Level 1) and between‐persons (Level 2). Each motives subtype and TAC drinking feature were partitioned into their between‐ and within‐person components prior to model estimation using R 4.3.2. Person‐mean enhancement, social, coping, and conformity motives, TAC rise rate, peak, and rise duration were used as between‐person predictors (Level 2); all were grand‐mean centered. Person‐mean centered enhancement, social, coping, and conformity motives, TAC rise rate, peak, and rise duration were used as within‐person predictors (Level 1). Predictors at the between‐ and within‐person levels (motives and TAC) were z‐scored prior to model estimation to allow for the comparison of effect sizes. Z‐score variables were created using the level‐specific standard deviations (e.g., between‐person SD for between‐person variables, within‐person SD for within‐person variables). All models included covariates for biological sex (0 = male, 1 = female) at Level 2, data collection burst at Level 1 (0 = burst 1, 1 = burst 2), and daily marijuana use at Level 1 (0 = no use, 1 = use). Covariates were centered in R prior to model estimation in MPlus. Models were also specified with a latent construct for TAC drinking features (three indicators of TAC intoxication: rise rate, peak, rise duration). Random intercepts and slopes were included in each model to allow the day‐level association between TAC intoxication and AIBs to differ between participants. Regressions on the TAC factor were modeled using linear paths because of its continuous nature (*M* = 0, SD = 1). AIBs were binary and modeled using a logit link and a binomial variance expectation. Bayesian models use Markov chain Monte Carlo sampling to generate a full posterior probability distribution of possible values for each parameter of interest. Our models used 10,000 iterations (50% warmup) across two chains to generate posterior distributions. Reported parameter estimates are the medians of posterior parameter distributions. Significance of parameter estimates was determined using 95% credibility intervals (CrI) of the posterior distributions (Makowski et al. 2019). Significant direct effects were determined by examining the 95% CrIs surrounding the parameter estimate of the paths (e.g., *path a2*, daily motives to daily TAC intoxication). Significant indirect effects were estimated directly by generating the product of posterior motives to TAC paths (*a paths*) and TAC to AIB paths (*b paths*) for each model iteration. The medians of ab posterior parameter distributions were reported as estimates of indirect effects; 95% CrIs were used to determine significance of indirect paths. The indirect and direct paths generate the total association of motives on AIBs (*c paths* = *a paths* x *b paths* + *c’ paths*). CrIs that did not contain the value of 0 for linear paths were considered statistically significant. Logistic paths were exponentiated to obtain odds ratios (OR), interpreted as a multiplicative increase (if OR > 1) or decrease (if OR < 1). CrIs that did not contain the value of 1 for logistic paths were considered statistically significant. Significance of indirect effects were also supplemented by the joint significance test (JST) (Leth‐Steensen and Gallitto 2016). The JST considers an indirect effect significant if both the component paths (e.g., path *a2* [predictor to mediator] and path *b2* [mediator to outcome]) are credibly significant. In all examined models, the JST agreed so we only report the product of the coefficients in the results. Sensitivity analyses were conducted examining the primary outcome in models from Aim 1 and Aim 2 using only morning reports of drinking motives. Supplemental models with fragmentary and en bloc AIBs as the outcome were also tested separately for Aim 1 and Aim 2. Findings from these models are presented in Table S1 through Table S6. Nearly all results were replicated, and all effects were in the same direction and of similar magnitude.

### 2.5 Transparency and Openness

We report how we determined our sample size, all data exclusions, all manipulations, and all measures in the study. Sample size was determined using Monte Carlo power simulations in MPlus. A sample size of 175 with 90% compliance had > 99% power to detect small effect sizes (0.1 SDs) at the within‐person level and 74% power (0.2 SDs) to detect small (0.2 SDs) or 96% power (0.3 SDs) to detect medium effect sizes at the between‐person level. Data and analytic code are publicly available through the NIMH Data Archive (https://doi.org/10.15154/xyp9‐5k89). The analyses of the current manuscript and the study design were not preregistered. An adapted version of the code used to process the Skyn data has been published (Richards and Russell 2025).

## 3 Results

Sensitivity analyses revealed that there were no associations between current drinking and prior day AIBs (OR = 1.21, 95% CI: 0.91, 1.59), fragmentary AIBs (OR = 1.23, 95% CI: 0.92, 1.61), or en bloc AIBs (OR = 1.56, 95% CI: 0.85, 2.53). Table 1 includes results from the fully unconditional models. The proportion of variance estimated at the within‐person level was highest for social motives (0.48), followed by enhancement (0.45), conformity (0.42), and coping (0.36). This suggests that drinking motives are not fixed characteristics and instead vary across days almost as much as they vary between persons. Results from the measurement model showed that all standardized loadings are above 0.4 and indicator R^2^ range from 23% to > 99% suggesting that the single factor solutions fit the data well (see Table 2).

**TABLE 1:** Results from “Empty” Unconditional Multilevel Models.

Model	Mean	Person‐level variance	Day‐level variance	Total variance^a^	ICC^b^	1−ICC^c^
Enhancement	0.82	0.23	0.19	0.42	0.55	0.45
Social	0.86	0.23	0.21	0.44	0.52	0.48
Coping	0.35	0.16	0.09	0.25	0.64	0.36
Conformity	0.14	0.05	0.04	0.09	0.58	0.42

^a^ Total variance calculated by summing the random intercept and the residual.

^b^ ICC calculated by dividing the fixed intercept by the total variance. This represents the proportion of variance observed at the between‐person level.

^c^ Calculated by subtracting the ICC from the value of 1. Represents the proportion of variance observed at the within‐person level.

**TABLE 2:** Factor Loadings Representing TAC Intoxication in Drinking Motive Models.

	Measurement		Aim 1								Aim 2	
			Enhancement		Social		Coping		Conformity		Single model	
Within‐person	Est.	95% CrI	Est.	95% CrI	Est.	95% CrI	Est.	95% CrI	Est.	95% CrI	Est.	95% CrI
Rise rate	0.85	0.84, 0.87	0.86	0.84, 0.87	0.86	0.84, 0.93	0.86	0.85, 0.88	0.86	0.85, 0.88	0.86	0.84, 0.87
Peak	1.00	1,00, 1.00	1.00	1.00, 1.00	1.00	0.99, 1.00	1.00	0.99, 1.00	1.00	0.99, 1.00	1.00	0.99, 1.00
Rise duration	0.48	0.44, 0.52	0.48	0.44, 0.52	0.49	0.45, 0.53	0.49	0.44, 0.53	0.49	0.45, 0.53	0.49	0.45, 0.53

Between‐person	Est.	95% CrI	Est.	95% CrI	Est.	95% CrI	Est.	95% CrI	Est.	95% CrI	Est.	95% CrI
Rise rate	0.90	0.86, 0.93	0.90	0.87, 0.93	0.90	0.87, 0.93	0.90	0.87, 0.93	0.90	0.87, 0.93	0.90	0.87, 0.93
Peak	1.00	0.98, 1.00	1.00	0.98, 1.00	1.00	0.98, 1.00	1.00	0.98, 1.00	1.00	0.98, 1.00	1.00	0.98, 1.00
Rise duration	0.59	0.48, 0.68	0.96	0.48, 0.69	0.59	0.48, 0.69	0.59	0.48, 0.69	0.60	0.48, 0.69	0.60	0.49, 0.70

*Note:* All factor loading estimates are standardized values.

Analyses of the study hypotheses include participants who self‐reported drinking alcohol on the morning or evening survey at least once during the 6 social weekends (*n* = 169, 1589 self‐reported drinking days, 97% of original sample retained). Of the 1589 self‐reported drinking days, there were 1373 (86.4%) corresponding days with positive TAC data. A total of 6 individuals from the original sample were removed from the analytic sample due to self‐reporting that they did not drink alcohol (*n* = 4) or not completing the daily surveys (*n* = 2). Days were excluded from the analyses if the participant had missing data on all predictor variables (*n* = 44). The final analytic sample resulted in 169 participants with 1545 self‐reported drinking days (see Table 3 for analytic sample demographics). About 80% of the sample experienced at least one AIB on days when they self‐reported that drinking occurred. In total, 535 AIBs were reported (*M* = 3.17; SD = 3.04). Participants reported a total of 532 fragmentary AIBs (*M* = 3.15, SD = 3.04) and a total of 90 en bloc AIBs (*M* = 0.53, SD = 1.19).

**TABLE 3:** Analytic Sample Participant Demographics (*N* = 169).

	*N*	%
**Age**		
18	5	3%
19	14	8.3%
20	39	23.1%
21	74	43.8%
22	26	15.4%
23	11	6.5%
**Biological sex**		
Female	90	53.3%
Male	79	46.7%
**Race**		
White	147	87%
Black or African American	6	3.6%
Asian	9	5.3%
Other	3	1.8%
Multiracial	4	2.4%
**Ethnicity**		
Not Hispanic or Latino	151	89.8%
Hispanic or Latino	17	10.1%
**Student status**		
Full Time	164	97%
Part Time	5	3%
**Year in school (full time students)**		
Freshman	12	7.3%
Sophomore	23	14%
Junior	49	29.9%
Senior	73	44.5%
Integrated (4 + 1 program)	2	1.2%
Graduate	5	3%

### 3.1 Aim 1

Table 2 shows factor loadings for the latent TAC intoxication variable. Factor loadings in each model examined for Aim 1 were similar at the within‐ and between‐person levels. Table 4 shows full results of the ML‐SEM testing the associations between drinking motives (separate models for each motive), TAC intoxication, and AIBs. At the within‐person level, the indirect effect was significant in all four models. On days when individuals reported higher drinking motives compared to themselves across days, they were at increased odds for experiencing an AIB, through its association with TAC intoxication (enhancement OR = 1.28; social OR = 1.29; coping OR = 1.15; conformity OR = 1.09). On days when individuals reported higher drinking motives compared to themselves across days, they experienced riskier TAC intoxication (enhancement *b* = 0.19; social *b* = 0.20; coping *b* = 0.11; conformity *b* = 0.07). Days with above average enhancement (OR = 1.17), social (OR = 1.27), and conformity motives (OR = 1.17) were directly associated with increased odds for experiencing an AIB, controlling for TAC intoxication. Coping motives were not directly associated with AIBs. Additional results from models testing fragmentary and en bloc AIBs as the outcome can be found in Tables S3 and S4.

**TABLE 4:** ML‐SEM Results for Individual Drinking Motive Models, TAC intoxication, and AIBs.

		Enhancement			Social			Coping			Conformity		
Within‐person	Paths	Est.	95% CrI		Est.	95% CrI		Est.	95% CrI		Est.	95% CrI	
TAC													
Motives	*a2*	**0.19**	**0.14**	**0.24**	**0.20**	**0.15**	**0.25**	**0.11**	**0.06**	**0.16**	**0.07**	**0.02**	**0.12**
Burst		0.02	−0.09	0.13	0.02	−0.09	0.13	−0.01	−0.12	0.11	−0.03	−0.14	0.08
Cannabis use		−0.17	−0.38	0.04	−0.19	−0.39	0.02	−0.22	−0.43	0.00	−0.19	−0.40	0.02
AIBs													
TAC	*b2*	**3.70**	**2.76**	**5.11**	**3.59**	**2.61**	**5.01**	**3.76**	**2.81**	**5.32**	**3.70**	**2.88**	**5.24**
Motives	*c2*	**1.17**	**1.01**	**1.36**	**1.27**	**1.10**	**1.47**	1.13	0.98	1.30	**1.17**	**1.01**	**1.35**
Burst		0.93	0.68	1.28	0.94	0.69	1.28	0.89	0.66	1.22	0.89	0.65	1.22
Cannabis use		1.34	0.75	2.43	1.36	0.76	2.42	1.35	0.74	2.45	1.32	0.72	2.38
Indirect effect													
Motives → TAC → AIB	*a2 x b2*	**1.28**	**1.18**	**1.41**	**1.29**	**1.18**	**1.42**	**1.15**	**1.07**	**1.25**	**1.09**	**1.02**	**1.18**
Total effect													
Motives → TAC → AIB	*a2 x b2 + c2*	**1.50**	**1.16**	**2.01**	**1.63**	**1.27**	**2.20**	**1.30**	**1.03**	**1.70**	**1.28**	**1.03**	**1.65**

Between‐person	Paths	Est.	95% CrI		Est.	95% CrI		Est.	95% CrI		Est.	95% CrI	
TAC													
Motives	*a1*	0.13	−0.04	0.30	0.12	−0.05	0.28	0.07	−0.11	0.24	0.09	−0.08	0.25
Sex		0.12	−0.22	0.46	0.11	−0.22	0.44	0.18	−0.25	0.41	0.10	−0.24	0.43
AIBs													
TAC	*b1*	**1.62**	**1.19**	**2.25**	**1.66**	**1.20**	**2.30**	**1.77**	**1.29**	**2.47**	**1.69**	**1.23**	**2.38**
Motives	*c1*	**2.19**	**1.66**	**2.95**	**2.03**	**1.53**	**2.74**	**1.55**	**1.16**	**2.12**	1.27	0.94	1.71
Sex		1.44	0.83	2.54	1.32	0.76	2.35	1.09	0.61	1.95	1.25	0.68	2.31
Indirect effect													
Motives → TAC → AIB	*a1 x b1*	1.06	0.98	1.19	1.05	0.98	1.18	1.04	0.94	1.17	1.04	0.96	1.16
Total effect													
Motives → TAC → AIB	*a1 x b1 + c1*	**2.33**	**1.64**	**3.76**	**2.15**	**1.51**	**3.46**	**1.61**	**1.13**	**2.63**	1.33	0.93	2.13

*Note:* Paths correspond to labels in theoretical model (see Figure 1). Covariate paths are blank as they were not pictured in the theoretical model. Estimates on paths predicting AIBs are the exponentiated, OR values. Bolded values indicate the estimate is credibly significant.

### 3.2 Aim 2

Factor loadings for the TAC intoxication variable were similar at the within‐ and between‐person levels (see Table 2). Table 5 shows full results of the single ML‐SEM testing the associations between each drinking motive, TAC intoxication, and AIBs. At the within‐person level, the only significant indirect effect observed was for social motives. On days when individuals reported higher social motives compared to themselves across days, they were at increased odds for experiencing an AIB, through its association with TAC intoxication (OR = 1.19). Enhancement, coping, and conformity motives did not have a significant indirect effect on AIBs. Social motives were the only motive to have a direct effect on TAC intoxication and AIBs. On days when individuals reported higher social motives, they experienced riskier TAC intoxication (*b* = 0.13) and had increased odds of experiencing an AIB (OR = 1.34). None of the other motives had a significant direct effect on TAC intoxication or AIBs. Additional results from models testing fragmentary and en bloc AIBs as the outcome can be found in Tables S5 and S6.

**TABLE 5:** ML‐SEM Results for Single Drinking Motives Model, TAC intoxication, and AIBs.

Within‐Person	Paths	Est.	95% CrI	
TAC				
Enhancement	*a5*	0.08	−0.01	0.17
Social	*a6*	**0.13**	**0.04**	**0.22**
Coping	*a7*	−0.01	−0.07	0.06
Conformity	*a8*	0.02	−0.04	0.07
Burst		0.02	−0.09	0.13
Cannabis Use		−0.18	−0.39	0.03
AIBs				
TAC	*b2*	**3.77**	**2.83**	**5.25**
Enhancement	*c5*	0.91	0.71	1.18
Social	*c6*	**1.34**	**1.04**	**1.73**
Coping	*c7*	0.99	0.84	1.18
Conformity	*c8*	1.12	0.97	1.31
Burst		0.92	0.67	1.26
Cannabis Use		1.33	0.73	2.44
Indirect Effect				
Enh → TAC → AIB	*a5 x b2*	1.11	0.99	1.26
Soc → TAC → AIB	*a6 x b2*	**1.19**	**1.06**	**1.36**
Cop → TAC → AIB	*a7 x b2*	0.99	0.91	1.08
Con → TAC → AIB	*a8 x b2*	1.02	0.95	1.10
Total Effect				
Enh → TAC → AIB	*a5 x b2 + c5*	1.02	0.70	1.57
Soc → TAC → AIB	*a6 x b2 + c6*	**1.59**	**1.09**	**2.50**
Cop → TAC → AIB	*a7 x b2 + c7*	0.99	0.78	1.30
Con → TAC → AIB	*a8 x b2 + c8*	1.15	0.93	1.47

Between‐Person	Paths	Est.	95% CrI	
TAC				
Enhancement	*a1*	0.16	−0.26	0.59
Social	*a2*	−0.01	−0.41	0.32
Coping	*a3*	−0.08	−0.34	0.17
Conformity	*a4*	0.08	−0.12	0.27
Sex		0.14	−0.20	0.49
AIBs				
TAC	*b1*	**1.63**	**1.19**	**2.24**
Enhancement	*c1*	**2.26**	**1.16**	**4.57**
Social	*c2*	1.08	0.55	2.06
Coping	*c3*	0.85	0.57	1.27
Conformity	*c4*	1.09	0.78	1.49
Sex		1.57	0.85	2.87
Indirect effect				
Enh → TAC → AIB	*a1 x b1*	1.07	0.88	1.41
Soc → TAC → AIB	*a2 x b1*	1.00	0.80	1.23
Cop → TAC → AIB	*a3 x b1*	0.97	0.83	1.09
Con → TAC → AIB	*a4 x b1*	1.03	0.94	1.17
Total effect				
Enh → TAC → AIB	*a1 x b1 + c1*	**2.44**	**1.11**	**7.37**
Soc → TAC → AIB	*a2 x b1 + c2*	1.08	0.52	2.66
Cop → TAC → AIB	*a3 x b1 + c3*	0.81	0.53	1.46
Con → TAC → AIB	*a4 x b1 + c4*	1.13	0.76	1.86

*Note:* Paths correspond to labels in the theoretical model (see Figure 2). Covariate paths are blank as they were not pictured in the theoretical model. Estimates on paths predicting AIBs are the exponentiated OR values. Bolded values indicate the estimate is credibly significant.

## 4 Discussion

The current study is the first to conduct a comprehensive examination of AIBs predicted by daily‐level prospective reports of drinking motives and TAC intoxication that was measured using a novel alcohol biosensor. TAC intoxication was represented by a latent biological intoxication factor capturing shared variance across three features of the ascending TAC curve: rise rate, peak, and rise duration. The strong and positive loadings of all three TAC features onto this factor support its interpretation as a composite measure of biological intoxication. Aim 1 examined separate models for each drinking motive to assess the raw, unadjusted contributions of each motive subtype on TAC intoxication and AIBs. Full support was observed for hypothesis 1a. In each individual motive model, on days when individuals reported higher enhancement, social, coping, and conformity motives compared to themselves across days, they experienced riskier TAC intoxication. Previous research at the day‐level is mixed regarding whether each motive subtype is associated with an increase in alcohol use. These findings support previous day‐level studies that suggest enhancement (Howard et al. 2024; Patrick and Terry‐McElrath 2021), social (Hamilton et al. 2020; Patrick and Terry‐McElrath 2021), coping (Dvorak et al. 2014; Hamilton et al. 2020), and conformity motives (Hamilton et al. 2020; O'Donnell et al. 2019) are positively associated with drinking. The results for conformity motives were particularly interesting. Conformity motives are not frequently endorsed among young adult samples (Boyle et al. 2026; Miller et al. 2020; O'Hara et al. 2014). Previous person‐level work has also suggested that conformity motives may be protective and result in decreased drinking (Bresin and Mekawi 2021; Cooper 1994). In contrast, O'Donnell et al. (2019) EMA examining drinking motives among young adults found that conformity motives were associated with both initiating a drinking episode and continuing to drink throughout the episode. Our findings are consistent with this work and suggest that conformity motives are not protective and may result in increased intoxication.

Partial support was observed for hypothesis 1b. Days with higher enhancement, social, and conformity motives directly increased the odds someone would experience an AIB. Coping motives did not have a significant direct association with AIBs. Previous person‐ and day‐level studies have inconsistently provided evidence for direct associations between motives and AIBs (e.g., Merrill and Read 2010; Merrill et al. 2014). When examining each motive individually, Ward et al. (2023) found significant positive within‐person associations for each drinking motive and AIBs. However, examining individual models, Merrill et al. (2019) found that only “to get high, buzzed, or drunk” (enhancement motive) was associated with AIBs. It is unclear why enhancement, social, and conformity motives would have a direct effect on AIBs after controlling for drinking. According to the Incentive Motivation Model of Alcohol Use, drinking motives may be impacted by substance use expectancies or context (Cox and Klinger 1988, 2011; Votaw and Witkiewitz 2021). It is possible there is a third unmeasured confounding variable like contextual risk or expectancies that would explain the direct effect between drinking motives and AIBs.

Full support was observed for each indirect effect hypothesis (H1c). On days when individuals reported higher motives relative to what they typically reported across days, they experienced riskier TAC intoxication, which in turn increased the odds of experiencing an AIB. The strength of the indirect effect was different in each model ([OR—1]*100). The indirect effect was strongest in the social motives model (29%), followed by enhancement (28%), coping (15%), and conformity (9%). Overall, findings from Aim 1 suggest that each motive subtype is a risk factor for increased intoxication and subsequent AIBs. The results from the conformity motives model are of particular interest. Previous work has suggested that conformity motives may be protective among not only drinking but also experiencing an AIB (Merrill et al. 2016). Findings from the current study highlight that conformity motives are a risk factor for both alcohol use and AIBs.

Aim 2 examined a single model with predictors for each motive subtype to test the independent contributions of each motive on TAC intoxication and AIBs. Partial support was observed for hypothesis 2a. When all four motives were included as predictors in a single model, the only motive significantly associated with TAC intoxication was social motives. Partial support was observed for hypothesis 2b. Social motives had a significant direct effect on AIBs, but enhancement, coping, and conformity motives did not. Finally, partial support was observed for hypothesis 2c. The only significant indirect effect observed was through the social motives path. Among young adults who reported the same levels of enhancement, coping, and conformity motives, days with higher social motives resulted in riskier TAC intoxication, which increased the odds someone would experience an AIB by 19%. We were not surprised to see that many of the motive subtype effects were reduced to non‐significance when examined in a single model. Previous work examining all motives as predictors of drinking and/or AIBs in a single model has found only one motive to emerge as significant (Merrill et al. 2014; Ward et al. 2023). Merrill et al. (2014) longitudinal study found that enhancement motives were the only motive subtype to indirectly impact AIBs through its association with drinking. Ward et al. (2023) found that coping motives were the only motive subtype associated with experiencing an AIB, controlling for baseline drinking, at the within‐person level. The current study differs from previous research by measuring drinking motives twice daily prior to alcohol use occurring, by using a biomarker to provide a precise measurement of intoxication that is measured in near‐real time, and by examining a sample of young adults who engage in risky drinking. Moreover, we explicitly tested drinking as a potential mechanism versus a covariate, which preserves the full association between motives and AIBs and allows elucidation of the potential pathways connecting them. Taken together, our findings suggest that (1) each drinking motive is important for the prediction of drinking and AIBs, but (2) only social motives offer predictive information above and beyond the other motives. Although the present study focused specifically on AIBs, AIBs are associated with experiencing more alcohol‐related consequences on days when they occur (Richards, Glenn, et al. 2023). Future research should consider examining whether the associations observed here extend to a broader range of alcohol‐related consequences.

### 4.1 Implications

Results have important implications for interventions focused on reducing risky drinking and AIBs. A primary focus of interventions for young adults is on increasing motivation to *change* drinking behaviors (Miller and Rollnick 2023). Brief Motivational Interventions (MI) utilize motivation to prompt behavior change (Larimer et al. 2022) and have been successful at reducing alcohol use and consequences (e.g., Cronce et al. 2018; Mun et al. 2023). Despite these successes, additional work needs to be done to strengthen brief MIs so that young adults' motivation for change is increased while simultaneously reducing drinking motives (Tan et al. 2023). An alternative approach for focusing on drinking motives are just‐in‐time adaptive interventions (JITAI) (Coughlin et al. 2021; Votaw and Witkiewitz 2021). JITAIs deliver interventions in the moment when they are needed to participants in their natural environment (Klasnja et al. 2015; Nahum‐Shani et al. 2018). Considering that each motive subtype is important for predicting drinking and AIBs, JITAI may be a beneficial intervention approach because they can offer personalized content to participants depending on which motive they endorse. There is also evidence suggest that JITAI are feasible, acceptable, and may be effective at reducing drinking motives (Blevins et al. 2021).

Research has also shown that drinking motives are moderated by protective behavioral strategies (Patrick et al. 2011; Schultz et al. 2024). Patrick et al. (2011) suggested that PBS may be important for experiencing fewer negative alcohol consequences among individuals who have higher coping and conformity motives. Schultz et al. (2024) found motives moderated associations between item‐level PBS items and alcohol‐related consequences. This suggested that drinking motives may be important for understanding the associations between PBS and negative alcohol consequences (Schultz et al. 2024). Future research may consider exploring how prospective daily‐level drinking motives impact TAC features and AIBs when above or below average PBS are used. JITAIs that aim to increase the use of PBS may also be effective at helping participants avoid AIBs on days when they report above average motives.

### 4.2 Limitations

The current study is not without limitations. First, participants were primarily undergraduate and graduate students recruited from a single large public university and identified primarily as White and Non‐Hispanic. Research shows that negative consequences from alcohol use differs by college attendance (e.g., 4‐year, 2‐year, vocational schools) and living situation (living at home with parents vs. living without parents on campus; Patrick et al. 2020). Findings may not be generalizable to non‐college attending young adults or students at smaller community colleges or vocational schools. Second, it is possible that TAC biosensors missed lower intensity drinking days (Barnett et al. 2014). There is currently no standard algorithm for detecting drinking days for the BACtrack Skyn biosensor (Richards et al. 2024). The guidelines used in the current study to detect drinking days were informed by previous studies (Courtney et al. 2023; Didier et al. 2024; Richards et al. 2024).

## 5 Conclusions

The current study contributes to the literature a prospective examination of daily drinking motives, TAC drinking features, and AIBs among a sample of young adults who engage in risky drinking. On days when individuals reported higher versus lower enhancement, social, coping, and conformity motives, they experienced riskier TAC intoxication, which subsequently increased the chances an AIB would occur. When all four drinking motives were examined as separate predictors in a single model, social motives were the only motive to impact AIBs through its association with TAC intoxication.

## Funding

This work was supported by National Institute on Alcohol Abuse and Alcoholism, F31AA031607, R21AA031528.

## Conflicts of Interest

The authors declare no conflicts of interest.

## Supporting information


**Table S1:** ML‐SEM Results for Individual Drinking Motive Models Using Only Morning Reports of Drinking Motives, TAC Intoxication, and AIBs.
**Table S2:** ML‐SEM Results for Single Drinking Motives Model Using Only Morning Reports of Drinking Motives, TAC Intoxication, and AIBs.
**Table S3:** ML‐SEM Results for Individual Drinking Motive Models, TAC Intoxication, and Fragmentary AIBs.
**Table S4:** ML‐SEM Results for Individual Drinking Motive Models, TAC Intoxication, and En Bloc AIBs.
**Table S5:** ML‐SEM Results for Single Drinking Motives Model, TAC Intoxication, and Fragmentary AIBs.
**Table S6:** ML‐SEM Results for Single Drinking Motives Model, TAC Intoxication, and En Bloc AIBs.

## Data Availability

The data that support the findings of this study are openly available in NIMH Data Archive at https://doi.org/10.15154/xyp9‐5k89.
