# Supplementary material for: Daily‐Level Drinking Motives as Predictors of Transdermal Alcohol Concentration and Alcohol‐Induced Blackouts Among Young Adults
Source: Alcohol Clin Exp Res (Hoboken). 2026 Oct 6;50(10):e70426. doi: 10.1111/acer.70426 (PMC13643198; doi:10.1111/acer.70426)
Supplement: Supplementary file 1 [file ACER-50-0-s001.docx]

| **Supplementary Table 1. ML-SEM Results for Individual Drinking Motive Models Using Only Morning Reports of Drinking Motives, TAC Intoxication, and AIBs.** | | | | | | | | | | | | | | |
| --- | --- | --- | --- | --- | --- | --- | --- | --- | --- | --- | --- | --- | --- | --- |
|  | |  | Enhancement | | | Social | | | Coping | | | Conformity | | |
| **Within-Person** | | **Paths** | **Est.** | **95% CrI** | | **Est.** | **95% CrI** | | **Est.** | **95% CrI** | | **Est.** | **95% CrI** | |
| TAC | |  |  |  |  |  |  |  |  |  |  |  |  |  |
|  | Motives | *a2* | **0.14** | **0.09,** | **0.20** | **0.17** | **0.12,** | **0.33** | **0.09** | **0.04,** | **0.14** | **0.07** | **0.02,** | **0.12** |
|  | Burst |  | 0.01 | -0.10, | 0.12 | 0.02 | -0.09, | 0.25 | -0.02 | -0.13, | 0.09 | -0.03 | -0.14, | 0.08 |
|  | Cannabis Use |  | -0.18 | -0.39, | 0.04 | -0.18 | -0.40, | 0.87 | -0.18 | -0.41, | 0.04 | -0.17 | -0.39, | 0.04 |
| AIBs | |  |  |  |  |  |  |  |  |  |  |  |  |  |
|  | TAC | *b2* | **3.65** | **2.81,** | **5.01** | **3.63** | **2.69,** | **5.05** | **3.65** | **2.75,** | **5.05** | **3.71** | **2.82,** | **5.23** |
|  | Motives | *c2* | 1.15 | 1.00, | 1.33 | **1.20** | **1.04,** | **1.39** | **1.17** | **1.02,** | **1.36** | **1.16** | **1.02,** | **1.34** |
|  | Burst |  | 0.93 | 0.68, | 1.27 | 0.94 | 0.69, | 1.29 | 0.91 | 0.67, | 1.23 | 0.88 | 0.65, | 1.21 |
|  | Cannabis Use |  | 1.34 | 0.74, | 2.48 | 1.31 | 0.72, | 2.39 | 1.29 | 0.71, | 2.34 | 1.31 | 0.72, | 2.35 |
| Indirect Effect | |  |  |  |  |  |  |  |  |  |  |  |  |  |
| Motives 🡪 TAC 🡪 AIB | | *a2 x b2* | **1.20** | **1.12,** | **1.31** | **1.25** | **1.15,** | **1.37** | **1.12** | **1.05,** | **1.22** | **1.09** | **1.02,** | **1.18** |
| Total Effect | |  |  |  |  |  |  |  |  |  |  |  |  |  |
| Motives 🡪 TAC 🡪 AIB | | *a2 x b2 + c2* | **1.39** | **1.09,** | **1.83** | **1.50** | **1.17,** | **2.38** | **1.32** | **1.06,** | **1.71** | **1.27** | **1.03,** | **1.63** |
| **Between-Person** | |  | **Est.** | **95% CrI** | | **Est.** | **95% CrI** | | **Est.** | **95% CrI** | | **Est.** | **95% CrI** | |
| TAC | |  |  |  |  |  |  |  |  |  |  |  |  |  |
|  | Motives | *a1* | 0.14 | -0.03, | 0.30 | 0.11 | -0.05, | 0.27 | 0.06 | -0.12, | 0.23 | 0.09 | -0.07, | 0.26 |
|  | Sex |  | 0.14 | -0.19, | 0.46 | 0.11 | -0.22, | 0.43 | 0.09 | -0.24, | 0.43 | 0.08 | -0.25, | 0.41 |
| AIBs | |  |  |  |  |  |  |  |  |  |  |  |  |  |
|  | TAC | *b1* | **1.60** | **1.19,** | **2.21** | **1.65** | **1.20,** | **2.28** | **1.74** | **1.25,** | **2.49** | **1.69** | **1.20,** | **2.45** |
|  | Motives | *c1* | **2.00** | **1.50,** | **2.72** | **1.95** | **1.48,** | **2.61** | **1.52** | **1.12,** | **2.08** | 1.20 | 0.89, | 1.64 |
|  | Sex |  | 1.42 | 0.81, | 2.50 | 1.27 | 0.71, | 2.28 | 1.11 | 0.61, | 2.02 | 1.23 | 0.67, | 2.25 |
| Indirect Effect | |  |  |  |  |  |  |  |  |  |  |  |  |  |
| Motives 🡪 TAC 🡪 AIB | | *a1 x b1* | 1.06 | 0.99, | 1.19 | 1.05 | 0.98, | 1.17 | 1.03 | 0.94, | 1.16 | 1.04 | 0.96, | 1.17 |
| Total Effect | |  |  |  |  |  |  |  |  |  |  |  |  |  |
| Motives 🡪 TAC 🡪 AIB | | *a1 x b1 + c1* | **2.14** | **1.49,** | **3.45** | **2.06** | **1.46,** | **3.27** | **1.57** | **1.09,** | **2.57** | 1.26 | 0.87, | 2.07 |
| ***Notes.*** *Paths correspond to labels in theoretical model (see Figure 1). Covariate paths are blank as they were not pictured in the theoretical model. Estimates on paths predicting AIBs are the exponentiated, OR values. Bolded values indicate the estimate is credibly significant.* | | | | | | | | | | | | | | |

| **Supplementary Table 2. ML-SEM Results for Single Drinking Motives Model Using Only Morning Reports of Drinking Motives, TAC Intoxication, and AIBs.** | | | | | | |
| --- | --- | --- | --- | --- | --- | --- |
| **Within-Person** | | | **Paths** | **Est.** | **95% CrI** | |
| TAC | | |  |  |  |  |
|  | Enhancement | | *a5* | 0.02 | -0.06, | 0.10 |
|  | Social | | *a6* | **0.15** | **0.07,** | **0.24** |
|  | Coping | | *a7* | 0.00 | -0.06, | 0.06 |
|  | Conformity | | *a8* | 0.02 | -0.04, | 0.08 |
|  | Burst | |  | 0.02 | -0.09, | 0.14 |
|  | Cannabis Use | |  | -0.19 | -0.40, | 0.02 |
| AIBs | | |  |  |  |  |
|  | TAC | | *b2* | **3.80** | **2.82,** | **5.34** |
|  | Enhancement | | *c5* | 0.97 | 0.76, | 1.23 |
|  | Social | | *c6* | 1.16 | 0.91, | 1.47 |
|  | Coping | | *c7* | 1.07 | 0.90, | 1.27 |
|  | | Conformity | *c8* | 1.11 | 0.96, | 1.30 |
|  | Burst | |  | 0.91 | 0.67, | 1.25 |
|  | Cannabis Use | |  | 1.33 | 0.72, | 2.43 |
| Indirect Effect | | |  |  |  |  |
| Enh 🡪 TAC 🡪 AIB | | | *a5 x b2* | 1.03 | 0.92, | 1.15 |
| Soc 🡪 TAC 🡪 AIB | | | *a6 x b2* | **1.22** | **1.09,** | **1.40** |
| Cop 🡪 TAC 🡪 AIB | | | *a7 x b2* | 1.00 | 0.92, | 1.09 |
| Con 🡪 TAC 🡪 AIB | | | *a8 x b2* | 1.03 | 0.95, | 1.11 |
| Total Effect | | |  |  |  |  |
| Enh 🡪 TAC 🡪 AIB | | | *a5 x b2 + c5* | 1.00 | 0.72, | 1.47 |
| Soc 🡪 TAC 🡪 AIB | | | *a6 x b2 + c6* | 1.42 | 0.97, | 2.19 |
| Cop 🡪 TAC 🡪 AIB | | | *a7 x b2 + c7* | 1.07 | 0.84, | 1.41 |
| Con 🡪 TAC 🡪 AIB | | | *a8 x b2 + c8* | 1.14 | 0.92, | 1.48 |
| **Between-Person** | | | **Paths** | **Est.** | **95% CrI** | |
| TAC | | |  |  |  |  |
|  | Enhancement | | *a1* | 0.27 | -0.18, | 0.71 |
|  | Social | | *a2* | -0.09 | -0.53, | 0.32 |
|  | Coping | | *a3* | -0.11 | -0.36, | 0.15 |
|  | Conformity | | *a4* | 0.09 | -0.11, | 0.29 |
|  | Sex | |  | 0.16 | -0.19, | 0.50 |
| AIBs | | |  |  |  |  |
|  | TAC | | *b1* | **1.64** | **1.18,** | **2.30** |
|  | Enhancement | | *c1* | **1.62** | **0.79,** | **3.39** |
|  | Social | | *c2* | 1.36 | 0.67, | 2.67 |
|  | Coping | | *c3* | 0.93 | 0.61, | 1.45 |
|  | Conformity | | *c4* | 0.96 | 0.67, | 1.35 |
|  | Sex | |  | 1.47 | 0.81, | 2.72 |
| Indirect Effect | | |  |  |  |  |
| Enh 🡪 TAC 🡪 AIB | | | *a1 x b1* | 1.13 | 0.92, | 1.53 |
| Soc 🡪 TAC 🡪 AIB | | | *a2 x b1* | 0.96 | 0.74, | 1.18 |
| Cop 🡪 TAC 🡪 AIB | | | *a3 x b1* | 0.96 | 0.81, | 1.08 |
| Con 🡪 TAC 🡪 AIB | | | *a4 x b1* | 1.04 | 0.95, | 1.18 |
| Total Effect | | |  |  |  |  |
| Enh 🡪 TAC 🡪 AIB | | | *a1 x b1 + c1* | **1.85** | **0.77,** | **6.15** |
| Soc 🡪 TAC 🡪 AIB | | | *a2 x b1 + c2* | 1.30 | 0.62, | 3.47 |
| Cop 🡪 TAC 🡪 AIB | | | *a3 x b1 + c3* | 0.89 | 0.57, | 1.65 |
| Con 🡪 TAC 🡪 AIB | | | *a4 x b1 + c4* | 1.00 | 0.66, | 1.71 |
| ***Notes.*** *Paths correspond to labels in theoretical model (see Figure 2). Covariate paths are blank as they were not pictured in the theoretical model. Estimates on paths predicting AIBs are the exponentiated, OR values. Bolded values indicate the estimate is credibly significant.* | | | | | | |

| **Supplementary Table 3. ML-SEM Results for Individual Drinking Motive Models, TAC Intoxication, and Fragmentary AIBs.** | | | | | | | | | | | | | | |
| --- | --- | --- | --- | --- | --- | --- | --- | --- | --- | --- | --- | --- | --- | --- |
|  | |  | Enhancement | | | Social | | | Coping | | | Conformity | | |
| **Within-Person** | | **Paths** | **Est.** | **95% CrI** | | **Est.** | **95% CrI** | | **Est.** | **95% CrI** | | **Est.** | **95% CrI** | |
| TAC | |  |  |  |  |  |  |  |  |  |  |  |  |  |
|  | Motives | *a2* | **0.19** | **0.14,** | **0.24** | **0.20** | **0.15,** | **0.25** | **0.10** | **0.05,** | **0.15** | **0.07** | **0.02,** | **0.12** |
|  | Burst |  | 0.01 | -0.10, | 0.12 | 0.02 | -0.09, | 0.12 | -0.02 | -0.13, | 0.09 | -0.03 | -0.14, | 0.08 |
|  | Cannabis Use |  | -0.17 | -0.38, | 0.04 | -0.19 | -0.40, | 0.02 | -0.20 | -0.41, | 0.02 | -0.20 | -0.42, | 0.02 |
| AIBs | |  |  |  |  |  |  |  |  |  |  |  |  |  |
|  | TAC | *b2* | **3.58** | **2.96,** | **5.32** | **3.81** | **2.85,** | **5.43** | **3.86** | **2.90,** | **5.32** | **3.76** | **2.82,** | **5.29** |
|  | Motives | *c2* | **1.18** | **1.02,** | **1.37** | **1.26** | **1.09,** | **1.47** | 1.15 | 0.99, | 1.32 | **1.17** | **1.02,** | **1.35** |
|  | Burst |  | 0.93 | 0.68, | 1.29 | 0.94 | 0.69, | 1.29 | 0.90 | 0.66, | 1.24 | 0.88 | 0.64, | 1.19 |
|  | Cannabis Use |  | 1.34 | 0.74, | 2.46 | 1.40 | 0.75, | 2.50 | 1.32 | 0.72, | 2.45 | 1.35 | 0.75, | 2.48 |
| Indirect Effect | |  |  |  |  |  |  |  |  |  |  |  |  |  |
| Motives 🡪 TAC 🡪 AIB | | *a2 x b2* | **1.28** | **1.18,** | **1.41** | **1.30** | **1.20,** | **1.46** | **1.15** | **1.07,** | **1.25** | **1.09** | **1.02,** | **1.18** |
| Total Effect | |  |  |  |  |  |  |  |  |  |  |  |  |  |
| Motives 🡪 TAC 🡪 AIB | | *a2 x b2 + c2* | **1.49** | **1.18,** | **2.03** | **1.65** | **1.27,** | **2.24** | **1.32** | **1.05,** | **1.71** | **1.27** | **1.03,** | **1.64** |
| **Between-Person** | |  | **Est.** | **95% CrI** | | **Est.** | **95% CrI** | | **Est.** | **95% CrI** | | **Est.** | **95% CrI** | |
| TAC | |  |  |  |  |  |  |  |  |  |  |  |  |  |
|  | Motives | *a1* | 0.13 | -0.04, | 0.30 | 0.11 | -0.05, | 0.27 | 0.07 | -0.11, | 0.24 | 0.09 | -0.08, | 0.25 |
|  | Sex |  | 0.12 | -0.21, | 0.45 | 0.10 | -0.22, | 0.43 | 0.09 | -0.25, | 0.42 | 0.10 | -0.24, | 0.43 |
| AIBs | |  |  |  |  |  |  |  |  |  |  |  |  |  |
|  | TAC | *b1* | **1.55** | **1.14,** | **2.15** | **1.56** | **1.14,** | **2.16** | **1.67** | **1.22,** | **2.32** | **1.62** | **1.18,** | **2.31** |
|  | Motives | *c1* | **2.22** | **1.66,** | **2.97** | **2.06** | **1.54,** | **2.77** | **1.55** | **1.15,** | **2.13** | 1.29 | 0.96, | 1.75 |
|  | Sex |  | 1.40 | 0.81, | 2.52 | 1.31 | 0.74, | 2.31 | 1.07 | 0.57, | 1.95 | 1.24 | 0.68, | 2.29 |
| Indirect Effect | |  |  |  |  |  |  |  |  |  |  |  |  |  |
| Motives 🡪 TAC 🡪 AIB | | *a1 x b1* | 1.05 | 0.98, | 1.18 | 1.04 | 0.98, | 1.16 | 1.03 | 0.94, | 1.15 | 1.04 | 0.96, | 1.15 |
| Total Effect | |  |  |  |  |  |  |  |  |  |  |  |  |  |
| Motives 🡪 TAC 🡪 AIB | | *a1 x b1 + c1* | **2.35** | **1.65,** | **3.74** | **2.16** | **1.53,** | **3.42** | **1.60** | **1.12,** | **2.61** | 1.35 | 0.95, | 2.15 |
| ***Notes.*** *Paths correspond to labels in theoretical model (see Figure 1). Covariate paths are blank as they were not pictured in the theoretical model. Estimates on paths predicting AIBs are the exponentiated, OR values. Bolded values indicate the estimate is credibly significant.* | | | | | | | | | | | | | | |

| **Supplementary Table 4. ML-SEM Results for Individual Drinking Motive Models, TAC Intoxication, and En Bloc AIBs.** | | | | | | | | | | | | | | | |
| --- | --- | --- | --- | --- | --- | --- | --- | --- | --- | --- | --- | --- | --- | --- | --- |
|  | |  | Enhancement | | | Social | | | Coping | | | Conformity | | |  |
| **Within-Person** | | **Paths** | **Est.** | **95% CrI** | | **Est.** | **95% CrI** | | **Est.** | **95% CrI** | | **Est.** | **95% CrI** | |  |
| TAC | |  |  |  |  |  |  |  |  |  |  |  |  |  |  |
|  | Motives | *a2* | **0.19** | **0.14,** | **0.24** | **0.20** | **0.14,** | **0.25** | **0.11** | **0.06,** | **0.16** | **0.06** | **0.01,** | **0.11** |  |
|  | Burst |  | 0.02 | -0.09, | 0.12 | 0.02 | -0.09, | 0.13 | -0.02 | -0.13, | 0.09 | -0.03 | -0.14, | 0.08 |  |
|  | Cannabis Use |  | -0.17 | -0.38, | 0.05 | -0.17 | -0.38, | 0.05 | -0.17 | -0.39, | 0.04 | -0.15 | -0.36, | 0.08 |  |
| AIBs | |  |  |  |  |  |  |  |  |  |  |  |  |  |  |
|  | TAC | *b2* | **1.74** | **1.02,** | **2.80** | 1.76 | 0.98, | 2.84 | **2.02** | **1.24,** | **3.22** | **1.82** | **1.08,** | **3.08** |  |
|  | Motives | *c2* | **1.41** | **1.09,** | **1.88** | **1.40** | **1.06,** | **1.86** | **1.28** | **1.02,** | **1.61** | **1.42** | **1.15,** | **1.78** |  |
|  | Burst |  | 1.25 | 0.69, | 2.29 | 1.22 | 0.68, | 2.22 | 1.17 | 0.65 | 2.16 | 1.09 | 0.60, | 2.01 |  |
|  | Cannabis Use |  | 1.12 | 0.36, | 3.30 | 1.12 | 0.36, | 3.36 | 1.02 | 0.32 | 2.99 | 0.98 | 0.31, | 2.79 |  |
| Indirect Effect | |  |  |  |  |  |  |  |  |  |  |  |  |  |  |
| Motives 🡪 TAC 🡪 AIB | | *a2 x b2* | **1.11** | **1.00,** | **1.23** | 1.11 | 1.00, | 1.25 | **1.08** | **1.02,** | **1.16** | **1.03** | **1.00,** | **1.10** |  |
| Total Effect | |  |  |  |  |  |  |  |  |  |  |  |  |  |  |
| Motives 🡪 TAC 🡪 AIB | | *a2 x b2 + c2* | **1.57** | **1.09,** | **2.41** | **1.57** | **1.05,** | **2.41** | **1.38** | **1.03,** | **1.94** | **1.47** | **1.16,** | **2.02** |  |
| **Between-Person** | |  | **Est.** | **95% CrI** | | **Est.** | **95% CrI** | | **Est.** | **95% CrI** | | **Est.** | **95% CrI** | |  |
| TAC | |  |  |  |  |  |  |  |  |  |  |  |  |  |  |
|  | Motives | *a1* | 0.13 | -0.04, | 0.30 | 0.13 | -0.04, | 0.29 | 0.07 | -0.10, | 0.24 | 0.08 | -0.08, | 0.25 |  |
|  | Sex |  | 0.12 | -0.21, | 0.45 | 0.09 | -0.26, | 0.41 | 0.08 | -0.26, | 0.41 | 0.08 | -0.25, | 0.41 |  |
| AIBs | |  |  |  |  |  |  |  |  |  |  |  |  |  |  |
|  | TAC | *b1* | **1.76** | **1.15,** | **2.91** | **1.75** | **1.12,** | **3.02** | **1.85** | **1.16,** | **3.23** | **1.68** | **1.10,** | **2.83** |  |
|  | Motives | *c1* | **2.45** | **1.52,** | **4.19** | **2.32** | **1.40,** | **4.11** | **1.88** | **1.16,** | **3.17** | **1.58** | **1.01,** | **2.61** |  |
|  | Sex |  | 1.06 | 0.39, | 2.72 | 1.01 | 0.37, | 2.60 | 0.76 | 0.25, | 2.11 | 0.89 | 0.33, | 2.31 |  |
| Indirect Effect | |  |  |  |  |  |  |  |  |  |  |  |  |  |  |
| Motives 🡪 TAC 🡪 AIB | | *a1 x b1* | 1.07 | 0.98, | 1.24 | 1.06 | 0.98, | 1.24 | 1.04 | 0.93, | 1.19 | 1.04 | 0.96, | 1.18 |  |
| Total Effect | |  |  |  |  |  |  |  |  |  |  |  |  |  |  |
| Motives 🡪 TAC 🡪 AIB | | *a1 x b1 + c1* | **2.64** | **1.51,** | **5.78** | **2.49** | **1.40,** | **5.65** | **1.96** | **1.14,** | **4.19** | 1.65 | 1.00, | 3.38 |  |
| ***Notes.*** *Paths correspond to labels in theoretical model (see Figure 1). Covariate paths are blank as they were not pictured in the theoretical model. Estimates on paths predicting AIBs are the exponentiated, OR values. Bolded values indicate the estimate is credibly significant.* | | | | | | | | | | | | | | | |

| **Supplementary Table 5. ML-SEM Results for Single Drinking Motives Model, TAC Intoxication, and Fragmentary AIBs.** | | | | | | |
| --- | --- | --- | --- | --- | --- | --- |
| **Within-Person** | | | **Paths** | **Est.** | **95% CrI** | |
| TAC | | |  |  |  |  |
|  | Enhancement | | *a5* | 0.07 | -0.02, | 0.16 |
|  | Social | | *a6* | **0.14** | **0.05,** | **0.22** |
|  | Coping | | *a7* | -0.01 | -0.07, | 0.06 |
|  | Conformity | | *a8* | 0.02 | -0.04, | 0.07 |
|  | Burst | |  | 0.02 | -0.09, | 0.13 |
|  | Cannabis Use | |  | -0.17 | -0.38, | 0.04 |
| AIBs | | |  |  |  |  |
|  | TAC | | *b2* | **3.92** | **2.89,** | **5.48** |
|  | Enhancement | | *c5* | 0.92 | 0.72, | 1.20 |
|  | Social | | *c6* | **1.31** | **1.02,** | **1.70** |
|  | Coping | | *c7* | 1.01 | 0.85, | 1.19 |
|  | | Conformity | *c8* | 1.12 | 0.96, | 1.30 |
|  | Burst | |  | 0.91 | 0.66, | 1.25 |
|  | Cannabis Use | |  | 1.31 | 0.72, | 2.44 |
| Indirect Effect | | |  |  |  |  |
| Enh 🡪 TAC 🡪 AIB | | | *a5 x b2* | 1.11 | 0.98, | 1.26 |
| Soc 🡪 TAC 🡪 AIB | | | *a6 x b2* | **1.20** | **1.06,** | **1.38** |
| Cop 🡪 TAC 🡪 AIB | | | *a7 x b2* | 0.99 | 0.91, | 1.08 |
| Con 🡪 TAC 🡪 AIB | | | *a8 x b2* | 1.03 | 0.95, | 1.11 |
| Total Effect | | |  |  |  |  |
| Enh 🡪 TAC 🡪 AIB | | | *a5 x b2 + c5* | 1.02 | 0.71, | 1.58 |
| Soc 🡪 TAC 🡪 AIB | | | *a6 x b2 + c6* | **1.58** | **1.07,** | **2.48** |
| Cop 🡪 TAC 🡪 AIB | | | *a7 x b2 + c7* | 1.00 | 0.79, | 1.31 |
| Con 🡪 TAC 🡪 AIB | | | *a8 x b2 + c8* | 1.15 | 0.93, | 1.48 |
| **Between-Person** | | | **Paths** | **Est.** | **95% CrI** | |
| TAC | | |  |  |  |  |
|  | Enhancement | | *a1* | 0.17 | -0.26, | 0.58 |
|  | Social | | *a2* | -0.01 | -0.40, | 0.40 |
|  | Coping | | *a3* | -0.08 | -0.34, | 0.17 |
|  | Conformity | | *a4* | 0.08 | -0.12, | 0.28 |
|  | Sex | |  |  |  |  |
| AIBs | | |  |  |  |  |
|  | TAC | | *b1* | **1.56** | **1.14,** | **1.65** |
|  | Enhancement | | *c1* | **2.43** | **1.23,** | **4.93** |
|  | Social | | *c2* | 1.01 | 0.50, | 1.95 |
|  | Coping | | *c3* | 0.85 | 0.56, | 1.29 |
|  | Conformity | | *c4* | 1.11 | 0.79, | 1.54 |
|  | Sex | |  | 1.58 | 0.86, | 2.95 |
| Indirect Effect | | |  |  |  |  |
| Enh 🡪 TAC 🡪 AIB | | | *a1 x b1* | 1.07 | 0.89, | 1.36 |
| Soc 🡪 TAC 🡪 AIB | | | *a2 x b1* | 1.00 | 0.81, | 1.21 |
| Cop 🡪 TAC 🡪 AIB | | | *a3 x b1* | 0.97 | 0.84, | 1.08 |
| Con 🡪 TAC 🡪 AIB | | | *a4 x b1* | 1.03 | 0.95, | 1.16 |
| Total Effect | | |  |  |  |  |
| Enh 🡪 TAC 🡪 AIB | | | *a1 x b1 + c1* | **2.61** | **1.18,** | **6.60** |
| Soc 🡪 TAC 🡪 AIB | | | *a2 x b1 + c2* | 1.00 | 0.47, | 2.38 |
| Cop 🡪 TAC 🡪 AIB | | | *a3 x b1 + c3* | 0.82 | 0.53, | 1.41 |
| Con 🡪 TAC 🡪 AIB | | | *a4 x b1 + c4* | 1.15 | 0.78, | 1.78 |
| ***Notes.*** *Paths correspond to labels in theoretical model (see Figure 2). Covariate paths are blank as they were not pictured in the theoretical model. Estimates on paths predicting AIBs are the exponentiated, OR values. Bolded values indicate the estimate is credibly significant.* | | | | | | |

| **Supplementary Table 6. ML-SEM Results for Single Drinking Motives Model, TAC Intoxication, and En Bloc AIBs.** | | | | | | |
| --- | --- | --- | --- | --- | --- | --- |
| **Within-Person** | | | **Paths** | **Est.** | **95% CrI** | |
| TAC | | |  |  |  |  |
|  | Enhancement | | *a5* | 0.08 | -0.01, | 0.17 |
|  | Social | | *a6* | **0.13** | **0.04,** | **0.22** |
|  | Coping | | *a7* | -0.01 | -0.07, | 0.06 |
|  | Conformity | | *a8* | 0.02 | -0.04, | 0.07 |
|  | Burst | |  | 0.02 | -0.10, | 0.12 |
|  | Cannabis Use | |  | -0.19 | -0.40, | 0.03 |
| AIBs | | |  |  |  |  |
|  | TAC | | *b2* | **1.87** | **1.09,** | **2.98** |
|  | Enhancement | | *c5* | 1.31 | 0.81, | 2.08 |
|  | Social | | *c6* | 1.02 | 0.64, | 1.69 |
|  | Coping | | *c7* | 1.02 | 0.76, | 1.37 |
|  | | Conformity | *c8* | **1.37** | **1.08,** | **1.74** |
|  | Burst | |  | 1.17 | 0.63, | 2.17 |
|  | Cannabis Use | |  | 1.09 | 0.35, | 3.54 |
| Indirect Effect | | |  |  |  |  |
| Enh 🡪 TAC 🡪 AIB | | | *a5 x b2* | 1.04 | 0.99, | 1.14 |
| Soc 🡪 TAC 🡪 AIB | | | *a6 x b2* | **1.08** | **1.01,** | **1.20** |
| Cop 🡪 TAC 🡪 AIB | | | *a7 x b2* | 1.00 | 0.95, | 1.04 |
| Con 🡪 TAC 🡪 AIB | | | *a8 x b2* | 1.01 | 0.97, | 1.05 |
| Total Effect | | |  |  |  |  |
| Enh 🡪 TAC 🡪 AIB | | | *a5 x b2 + c5* | 1.37 | 0.81, | 2.50 |
| Soc 🡪 TAC 🡪 AIB | | | *a6 x b2 + c6* | 1.11 | 0.64, | 2.15 |
| Cop 🡪 TAC 🡪 AIB | | | *a7 x b2 + c7* | 1.01 | 0.75, | 1.45 |
| Con 🡪 TAC 🡪 AIB | | | *a8 x b2 + c8* | **1.38** | **1.08,** | **1.88** |
| **Between-Person** | | | **Paths** | **Est.** | **95% CrI** | |
| TAC | | |  |  |  |  |
|  | Enhancement | | *a1* | 0.13 | -0.29, | 0.57 |
|  | Social | | *a2* | 0.02 | -0.39, | 0.43 |
|  | Coping | | *a3* | -0.07 | -0.33, | 0.18 |
|  | Conformity | | *a4* | 0.07 | -0.13, | 0.27 |
|  | Sex | |  | 0.13 | -0.22, | 0.47 |
| AIBs | | |  |  |  |  |
|  | TAC | | *b1* | **1.75** | **1.09,** | **2.89** |
|  | Enhancement | | *c1* | 2.26 | 0.68, | 7.63 |
|  | Social | | *c2* | 1.06 | 0.33, | 3.50 |
|  | Coping | | *c3* | 1.01 | 0.48, | 1.99 |
|  | Conformity | | *c4* | 1.36 | 0.79, | 2.35 |
|  | Sex | |  | 0.96 | 0.35, | 2.73 |
| Indirect Effect | | |  |  |  |  |
| Enh 🡪 TAC 🡪 AIB | | | *a1 x b1* | 1.06 | 0.83, | 1.49 |
| Soc 🡪 TAC 🡪 AIB | | | *a2 x b1* | 1.01 | 0.78, | 1.32 |
| Cop 🡪 TAC 🡪 AIB | | | *a3 x b1* | 0.97 | 0.80, | 1.12 |
| Con 🡪 TAC 🡪 AIB | | | *a4 x b1* | 1.03 | 0.93, | 1.20 |
| Total Effect | | |  |  |  |  |
| Enh 🡪 TAC 🡪 AIB | | | *a1 x b1 + c1* | 2.43 | 0.66, | 13.98 |
| Soc 🡪 TAC 🡪 AIB | | | *a2 x b1 + c2* | 1.07 | 0.32, | 5.49 |
| Cop 🡪 TAC 🡪 AIB | | | *a3 x b1 + c3* | 0.97 | 0.47, | 2.41 |
| Con 🡪 TAC 🡪 AIB | | | *a4 x b1 + c4* | 1.42 | 0.78, | 3.13 |
| ***Notes.*** *Paths correspond to labels in theoretical model (see Figure 2). Covariate paths are blank as they were not pictured in the theoretical model. Estimates on paths predicting AIBs are the exponentiated, OR values. Bolded values indicate the estimate is credibly significant.* | | | | | | |
